# Antibacterial, antibiofilm, and anti-quorum sensing activities of pyocyanin against methicillin-resistant *Staphylococcus aureus*: in vitro and in vivo study

**DOI:** 10.1186/s12866-023-02861-6

**Published:** 2023-04-24

**Authors:** Amal M. Abo Kamer, Ahmed A. Abdelaziz, Khaled B. Al-Monofy, Lamiaa A. Al-Madboly

**Affiliations:** Department of Pharmaceutical Microbiology, Faculty of Pharmacy, Tanta University, Tanta, Egypt

**Keywords:** MRSA, Antibacterial, Pyocyanin, Antibiofilm, Anti-Quorum sensing, Antivirulence agent

## Abstract

**Background:**

Methicillin-resistant *Staphylococcus aureus* (MRSA) infections are considered a major public health problem, as the treatment options are restricted. Biofilm formation and the quorum sensing (QS) system play a pivotal role in *S. aureus* pathogenicity. Hence, this study was performed to explore the antibacterial effect of pyocyanin (PCN) on MRSA as well as its effect on MRSA biofilm and QS.

**Results:**

Data revealed that PCN exhibited strong antibacterial activity against all test MRSA isolates (*n* = 30) with a MIC value equal to 8 µg/ml. About 88% of MRSA biofilms were eradicated by PCN treatment using the crystal violet assay. The disruption of MRSA biofilm was confirmed using confocal laser scanning microscopy, which showed a reduction in bacterial viability (approximately equal to 82%) and biofilm thickness (approximately equal to 60%). Additionally, the disruption of the formation of microcolonies and the disturbance of the connection between bacterial cells in the MRSA biofilm after PCN treatment were examined by scanning electron microscopy. The 1/2 and 1/4 MICs of PCN exerted promising anti-QS activity without affecting bacterial viability; Agr QS-dependent virulence factors (hemolysin, protease, and motility), and the expression of *agrA* gene, decreased after PCN treatment. The in silico analysis confirmed the binding of PCN to the AgrA protein active site, which blocked its action. The in vivo study using the rat wound infection model confirmed the ability of PCN to modulate the biofilm and QS of MRSA isolates.

**Conclusion:**

The extracted PCN seems to be a good candidate for treating MRSA infection through biofilm eradication and Agr QS inhibition.

**Supplementary Information:**

The online version contains supplementary material available at 10.1186/s12866-023-02861-6.

## Background

Antibiotic resistance has become a universal public health problem. At least 700,000 people per year die from antibiotic-resistant infections, and it is predicted that this figure will increase to 10 million by 2050, making bacterial antimicrobial resistance (AMR) a global health problem [1–3]. *Staphylococcus aureus* is one of the most prevalent pathogens in the community and hospital-acquired infections [4]. In Europe, 16.9% of hospital-acquired infections in 2017 were caused by methicillin-resistant *S. aureus* (MRSA) [3]. Even though *S. aureus* is known to be a part of the normal skin microbiota, it is commonly the cause of infections, including those of the skin, soft tissues, bloodstream, and respiratory tract [5]. MRSA displays an unexpected capability to develop resistance to recently improved antibiotics [6]. Therefore, finding new medications to treat infections caused by MRSA is as a great priority [7].

One of the factors that makes *S. aureus* infections more difficult to treat is their ability to develop biofilms. As explored by researchers, these structures are considered a clinical challenge because they are extremely resistant to antimicrobial agents and host defenses [8]. Biofilm acts as a physical barrier, preventing the penetration of antibiotics and their interaction with bacterial cells, as well as protecting bacteria from innumerable environmental pressures [9]. Therefore, targeting biofilm in the treatment of *S. aureus* infections is one of the most effective approaches for diminishing the occurrence of resistance.

Microbial cells within the biofilm communicate with each other through quorum sensing (QS), which adjusts metabolic activity and promotes virulence [10]. In *S. aureus*, the virulence factors, such as hemolysin, protease, and motility, are directly and indirectly under the control of Agr-mediated QS [11–13]. Blocking QS disarms the pathogen from several virulence factors as well as lowering its ability to colonize and invade host tissue [14–16]. Hence, antivirulence agents targeting QS represent a promising strategy for treating MRSA infections.

Pyocyanin (PCN), a great research pigment produced by 90–95% of *Pseudomonas aeruginosa* isolates, simply diffuses through and permeates cell membranes, where it produces reactive oxygen species (ROS) [17]. In prokaryotes, the ability of PCN to kill microorganisms is mediated via the production of ROS, which results in the induction of oxidative stress and the inhibition of the ion's interaction with the membrane, respiration, and the active transport of solutes [18, 19]. In light of this scenario, the growth inhibition, the biofilm eradication, and the QS attenuation activities of PCN against MRSA isolates were assessed in our study using in vitro and in vivo approaches.

In conclusion, PCN is an active agent for growth inhibition and biofilm eradication of MRSA isolates. Moreover, PCN exerts a remarkable anti-QS effect by targeting Agr QS. Nevertheless, more investigations are essential to carry out clinical trials of PCN in the future.

## Methods

### Preparation of PCN

The laboratory extraction, purification, and characterization of PCN as well as cytotoxicity evaluation were discussed in our previous study [20]. In the present study, the sub-toxic concentrations of PCN (less than 45 µg/ml) were used for evaluation of the antibacterial, antibiofilm, and anti-QS activities of PCN against MRSA isolates.

### Bacterial strains

Bacterial samples from different sources (blood (23), wound (78), sputum (26), and abscess (33)) were first isolated from patients admitted to Tanta University Hospital in Egypt who suffered from bacteremia, skin and soft tissue infections, and pulmonary infections. The isolated samples were cultured in nutrient broth and streaked on nutrient agar plates. Separated bacterial colonies were exposed to conventional identification steps, including the Gram-staining technique, growth on mannitol salt agar plates, and biochemical identification, as prescribed by [21]. *Staphylococcus aureus* ATTC 25,913 was used as a reference strain. Isolates identified as *S. aureus* (*n* = 100) were preserved in tryptic soy broth containing 10% (v/v) glycerol at − 80 °C for further studies.

### Susceptibility pattern determination

The collected *S. aureus* isolates were subjected to the Kirby-Bauer disk diffusion method using oxacillin (OX; 1 μg), and cefoxitin (FOX; 30 μg) for the determination of MRSA isolates from collected isolates, as prescribed by [22]. The antimicrobial susceptibility testing for the obtained MRSA isolates was conducted using Mueller–Hinton agar (MHA) plates, according to CLSI 2018. The following antimicrobials were tested, penicillin (P; 10 μg), azithromycin (AZM; 15 μg), chloramphenicol (C; 30 μg), gentamicin (GN; 10ug), trimethoprim-sulfamethoxazole (SXT; 1.25/23.75 μg), erythromycin (E; 15 μg), linezolid (LZD; 30 μg), levofloxacin (LEV; 5 μg), tetracycline (T; 30 μg), clindamycin (CC; 2 μg), rifampicin (RA; 5 μg) and vancomycin (V; 30 μg).

### Antibacterial activity of PCN

A well-diffusion approach, described by [23] with a few modifications, was used to test the antibacterial activity of PCN against MRSA isolates. Briefly, 6 mm-diameter wells were punched through MHA plates. On the surface of the MHA plates, about 100 µl of each tested microorganism (0.5 McFarland) was distributed evenly. Each well received a different concentration of PCN (10, 20, and 40 µg/ml), which was made by weighing and dissolving PCN powder in sterilized distilled water (SDW). The antibacterial activity was measured in terms of the diameter of the zone of inhibition (mm) developed around each well after the plates were incubated at 37 °C for 24 h, and SDW served as a negative control.

### The Minimum Inhibitory Concentration of PCN (MIC)

According to the CLSI 2018 standard methodology, the broth microdilution assay in MH broth was assessed to evaluate MIC values (1–512 µg/ml) of PCN against MRSA isolates. After overnight incubation at 37 °C, the microplates were investigated against a black background, and the growth of the tested microorganism was evaluated according to the turbidity of the broth. The lowest concentration of PCN that visually showed no growth was determined as MIC [24].

### Biofilm formation and virulence factors determination

Biofilm formation and virulence factors (proteolytic activity, hemolytic activity, and motility) of MRSA isolates were screened for the identification of the strongest biofilm-forming and most virulent strains. The biofilm-forming ability was evaluated, as described in [25]. Briefly, MRSA strains were cultured in 96-well plates containing MHB media and 1% glucose for 24 h at 37 °C. The cultures were removed, and the plates were washed twice with phosphate-buffered saline (PBS). The resulting biofilm was stained with 0.1% crystal violet for 15 min, and the excess stain was removed by washing with PBS. The remained biofilm was solubilized by 33% (vol/vol) glacial acetic acid and quantified by measuring absorbance at 595 nm using a microtiter reader (Sunrise™, TECAN, Switzerland). For proteolytic activity, MRSA strains were inoculated on skim milk agar plates (5% skimmed milk in 1.5% LB agar). After 48 h of incubation at 28 °C, plates were observed to evaluate the formation of lysis zones around the inoculated bacteria [26]. Hemolytic activity was verified through inoculation of MRSA strains in LB agar with 4% human blood. After 48 h of incubation at 28 °C, plates were observed to evaluate the formation of lysis zones around the inoculated bacteria [27]. For motility, MRSA strains were stab-inoculated deep inside semi-solid agar (0.3% meat extract, 0.5% peptone, and 0.5% agar). After 3 days of incubation at 37 °C, non-motile bacteria remain near the inoculation site, and motile bacteria spread and visibly cloud the media [28].

### Effect of PCN on established biofilm

The effect of PCN on the developed biofilms of MRSA was estimated using the method described by [29]. About 200 µl of the test bacterial culture (10^6^ CFU/ml) was transferred to a 96-well microtiter plate. The 48 h pre-established biofilms were treated with PCN at different concentrations (10, 20, and 40 µg/ml), and untreated wells served as a positive control. After 24 h of incubation at 37 °C, the plates were washed twice with PBS. The resulting biofilm was stained with 0.1% crystal violet for 15 min, and the excess strain was removed by washing with PBS. After solubilization of the remaining stain with 33% (vol/vol) glacial acetic acid, the microplate was evaluated by a microplate reader (Sunrise™, TECAN, Switzerland) using spectrophotometric measurements at 595 nm. The formula used to get the percentage of eradication was [(OD (control)-OD (test)/OD (control)) × 100]. The biofilm eradication activity of PCN was further confirmed using a light microscope (LM), as described by [25, 30]. In brief, 1 ml of the inoculated broth (MHB media containing 1% glucose) having a concentration of 10^8^ CFU/ml was transferred to a 6-well microtiter plate containing a 1 × 1 cm size of coverslips. After 48 h of incubation at 37 °C, planktonic cells were removed, and the wells were gently washed with normal saline three times. Thereafter, 500 µl of PCN (10, 20, and 40 µg/ml) was added. Later, the entire plate was incubated at 37 °C for 24 h under static conditions. After incubation, glass coverslips were removed, washed with PBS, and stained with 0.1% crystal violet. After the removal of excessive stains by washing with deionized water and drying, the coverslips were examined at 100 × magnification using LM (LABOMED, CXL, USA).

### Determination of Colony Forming Unit (CFU)

To determine the effect of PCN on bacterial viability within pre-established MRSA biofilms, we determined the count of viable bacterial cells in the presence and absence of PCN, as previously described by [31]. Briefly, after the biofilm formation of MRSA isolates using the microtiter plate method, as mentioned above, in the presence and absence of PCN, the wells of the microtiter plates were washed twice using PBS for the removal of lightly attached bacterial cells. Then, biofilms were scraped from the wells using a pipette tip, and 200 μl of PBS was added to each well, followed by homogenizing the biofilms using the vortex. Finally, the homogenized biofilm was successively diluted using PBS, and 100 μl from each individual dilution was plated on MHA plates. The CFU was counted after overnight incubation at 37 °C.

### Quantification of exopolysaccharide (EPS)

The effect of PCN on EPS production was assessed using an approach previously described by [32]. In brief, the MRSA isolates were incubated in LB broth with and without PCN at a concentration of 2 and 4 µg/ml. After incubation for 24 h at 37 °C, centrifugation at 8000 × g for 10 min was done, and pellets were suspended using PBS and then centrifuged again. The obtained supernatant was mixed with an equal volume of ethyl alcohol and centrifuged. Lastly, the EPS solution (1 ml) was carefully mixed with cold 5% phenol (1 ml) and concentrated sulfuric acid (5 ml). The percentage of reduction of EPS after PCN treatment was calculated from the measured optical density at 490 nm of the resultant red color.

### Scanning Electron Microscope analysis (SEM)

The antibiofilm potential of PCN on the preformed MRSA biofilm was visualized under SEM, as prescribed by [33]. In brief, the biofilms were developed on 1 × 1 cm size coverslips with all procedures, as described above. After biofilm fixation using 2.5% glutaraldehyde at 37 °C for 30 min, coverslips were washed three times with PBS solution and dehydrated using ethanol. Finally, biofilms were successively dehydrated, air-dried, sputter-coated with gold (Hitachi®, Tokyo, Japan), and examined under SEM (S-34002N SEM, Hitachi®, Tokyo, Japan).

### Confocal Laser Scanning Microscopy (CLSM)

The antibiofilm potential of PCN on the preformed MRSA biofilm was visualized under a CLSM, as prescribed by [34]. The 8-well chamber slide (ibidi, Martinsried, Germany), on which biofilms developed, was washed three times with PBS to remove the planktonic cells. After adding 5 μL acridine orange (green fluorescence), which stains live cells, and 5 μL propidium iodide (red fluorescence), which stains dead cells, for 15 min in the dark, the structure of the biofilm was examined by CLSM (DMi8; Leica Microsystem).

### Effect of PCN on growth curve

The most virulent MRSA strains (*n* = 10) were cultured in LB broth with an OD_600_ of 0.3 with 3/4, 1/2, and 1/4 MICs of PCN and without PCN at 37 °C. Samples were collected and the absorbance was read at 600 nm at 30-min intervals; 3 ml samples of each culture were collected immediately after the addition of PCN (zero time) and after 30, 60, 90, 120, 150, 180, 210, 240, 270, 300, 330, 360, and 420 min [35]. The sub-MIC concentrations of PCN that did not affect growth were used for the subsequence experiments.

### Effect of PCN on protease production

Overnight cultures of each tested MRSA strain in LB broth with and without 2 and 4 µg/ml PCN were centrifuged and then filtered using a 0.45 μm filter. Sterile supernatants (100 μL) were added to the wells made in the skim milk agar plates. Clear zones that formed around the wells after 24 h of incubation at 37 °C were measured [36].

### Effect of PCN on hemolysin production

Qualitative and quantitative approaches were utilized for the detection of the effect of PCN on hemolysin production. In the qualitative approach, the overnight culture of a bacterial strain (20 μL) was inoculated into LB broth (180 μl) with or without PCN (4 μg/ml). After incubating at 37 °C for 18 h, the samples were streaked on human blood agar. The zone of red cell clearance around inoculated bacteria was observed after 24 h [37]. In the quantitative approach, MRSA strains were inoculated in LB broth with and without 2 and 4 µg/ml PCN. After centrifugation and filtration of supernatants, we combined 600 μL of a 2% suspension of red blood cells (RBCs) with 600 μL of supernatant and incubated this mixture for 2 h at 37 °C. The suspension was centrifuged at 10,000 g for 8 min at 4 °C, and hemoglobin release was measured by determining absorbance at 540 nm [38].

### Effect of PCN on motility

The motility assay was conducted using motility plates alone and motility plates containing 2 and 4 µg/ml PCN. The plates were inoculated with a sterile toothpick and incubated at 37 °C for 24 h. Motility was assessed by measuring the zone formed by the colonies migrating away from the point of inoculation [39].

### Gene expression analysis

The most virulent MRSA strains (*n* = 10) were cultured in LB broth with and without PCN (4 µg/ml). The kit manufacturer’s instructions (Roche Diagnostic GmbH, Germany) were followed for extracting total RNA from bacterial pellets. The RNA yield and purity were evaluated by measuring the absorbance, and samples with a ratio of 260/280 nm in the 1.8–2 range were used. Synthesis of cDNA was conducted according to the kit manufacturer’s instructions (ThermoFisher Scientific, Waltham, MA, USA). The qRT-PCR was performed using a RT-PCR device, Rotor-Gene Q (Qiagen, USA), to determine transcript levels of genes using oligonucleotides listed in Table 1. For housekeeping genes, the *16S rRNA* gene was used, and the cycling conditions were 94 °C for 3 min, followed by 30 cycles of denaturation at 94 °C for 30 s, annealing at 60 °C for 30 s, extension at 72 °C for 30 s, and a final extension cycle of 5 min at 72 °C [3].

**Table 1:** List of primers used in qRT-PCR experiment

Genes	Oligonucleotides
*16S rRNA*	F: AAACTCAAAKGAATTGACGG R: CTCACRRCACGAGCTGAC
*agrA*	F: GCACATACACGCTTACAATTGTTG R: ACACTGAATTACTGCCACGTTTTAAT

### Molecular docking analysis

The binding interaction between PCN and the AgrA protein was performed by the Molecular Operating Environment (MOE, 10.2008) software. The crystal protein structure of the AgrA protein of *S. aureus* (PDB ID: 3BS1) was downloaded from the Protein Data Bank (http://www.rcsb.org/). Ligands and water molecules not implicated in the binding were detached. Then, the ArgA protein was ready for docking analysis using the protonate 3D protocol in MOE with default options. The PCN structure was drawn in ChemBioDraw (version 14) software [11].

### Wound infection model

The following procedures were permitted by the Faculty of Pharmacy-Tanta University Research Ethics Committee (TP/RE/12/22p-0068), and the study was conducted in accordance with ARRIVE guidelines. Male BALB/c rats of 120–150 g in weight (*n* = 12) were obtained from 6 to 8 weeks of age from the College of Veterinary Medicine of Cairo University (Cairo, Egypt) animal house. After wound creation, rats were individually housed to prevent cross-contamination and fighting. Rats were randomly divided into two groups (*n* = 6); group 1 was injected with vehicle (PBS) only, while group 2 received PCN dissolved in the vehicle at a concentration of 40 μg/ml. The procedures were established as previously performed by [40, 41], and the rats were permitted to correct the environment before starting the experiments. Each rat was individually maintained in a ventilated cage with 12 h of light and 12 h of darkness at room temperature and unrestricted contact with food and water. Ketamine (40 mg kg − 1) and xylazine (5 mg kg − 1)-anesthetized rats were shaved from the back and sterilized using 10% povidone-iodine. Two excisional wounds, each 10 mm in thickness, were performed on the dorsal section of the rat using biopsy punches on each lateral of the spine. The established wounds were infected with 10 μL of the bacterial suspension (10^6^ CFU) of the most virulent MRSA isolate. Following inoculation, 30 min later, 20 μL of the vehicle (PBS) was injected subcutaneously in the control group (group 1), and PCN was injected subcutaneously in the treated group (group 2) on days 0, 3, and 6. Images of wounds were taken by the camera. Wound size was quantified by analysis using Image J software version 146. Wound contractions were presented as a percentage of the wounded area according to the following formula: percentage of wound closure = ((initial wound size—wound at the time of taking the image)/initial wound size) × 100. At the end of our experiment, six animals from each group were euthanized by CO_2_ inhalation on day 6. The skin lesions were excised with 2–5 mm of the area and collected for histopathological examination using hematoxylin and eosin (H & E) at the Pathology Department, Faculty of Medicine, Tanta University, and for the counting of bacterial load at the wound area. After the homogenization of tissues by repeated application of a 1 ml syringe plunger in a microfuge tube that contained the tissue and distilled PBS, the homogenized suspensions were serially diluted and plated on mannitol salt agar for CFU count determination [42]. The bacterial burden (CFU per gram) of tissue was calculated by the following equation: CFU per gram = (plate count × $\frac{1}{dilution}$  × 10)/weight of homogenized tissue.

### Statistical analysis

The experiments were performed in triplicate, and the data were expressed as mean ± SD. A T-test was used to compare the two groups. The data of more than two groups were analyzed by one-way analysis of variance (ANOVA) using GraphPad Prism version 5 software, and the threshold for significance was set at a *p*-value < 0.05.

## Results

### Bacterial test isolates and susceptibility pattern

Based on culture on mannitol salt agar plates, Gram-staining technique, and the catalase test, *S. aureus* (*n* = 100) was isolated from different specimens (blood (10), wound (60), sputum (12), and abscess (18)). The percentage of MRSA isolates (resistant to both oxacillin and cefoxitin) was 30%. The percentage of antimicrobial resistance of MRSA strains involved in the study (*n* = 30) was the following: penicillin (80%), aztreonam (43.3%), chloramphenicol (40%), gentamycin (73.3%), rifampin (0%), vancomycin (0%), trimethoprim-sulfamethoxazole (43.3%), erythromycin (83.3%), linezolid (0%), levofloxacin (56.6%), tetracycline (36.6%), clindamycin (33.3%), oxacillin (100%) and cefoxitin (100%) as shown in Table 2.

### Biofilm formation and virulence determination

Biofilm formation and virulence factors of MRSA isolates, such as protease, hemolysin, and motility, were estimated for detecting the strongest biofilm-forming and most virulent MRSA isolates. These MRSA isolates were used for evaluating the effect of PCN on the eradication of their pre-established biofilm and the production of their virulence factors. From Table 3, the strongest biofilm-forming and most virulent MRSA strains were MRSA Nos. 1, 2, 3, 14, 16, 22, 24, 25, 27, and 30.

**Table 2:**
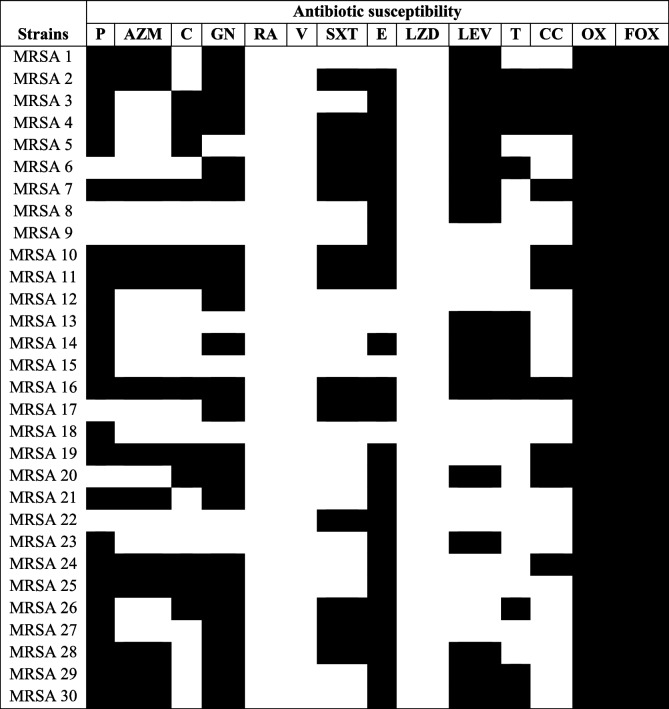
Resistance pattern of MRSA strains (*n* = 30). Black areas in the resistance pattern refer to resistance to antibiotics, and white areas in the resistance pattern refer to sensitivity to antibiotics. P; penicillin (10 μg), AZM; azithromycin (15 μg), C; chloramphenicol (30 μg), GN; gentamicin (10ug), RA; rifampicin (5 μg), V; vancomycin (30 μg), SXT; trimethoprim-sulfamethoxazole (1.25/23.75 μg), E; erythromycin (15 μg), LZD; linezolid (30 μg), LEV; levofloxacin (5 μg), T; tetracycline (30 μg), CC; clindamycin (2 μg), OX; oxacillin (1 μg), FOX; cefoxitin (30 μg)

**Table 3:** Virulence factors of MRSA strains (*n* = 30). + indicates the presence of the virulence factor, − indicates the absence of the virulence factor, SBF indicates strong biofilm formation, MBF indicates moderate biofilm formation, and WBF indicates weak biofilm formation

Strains	Virulence factors			
	Biofilm	Protease	Hemolysin	Motility
MRSA 1	SBF	** + **	** + **	** + **
MRSA 2	SBF	** + **	** + **	** + **
MRSA 3	SBF	** + **	** + **	** + **
MRSA 4	SBF	** + **	** − **	** + **
MRSA 5	SBF	** + **	** − **	** − **
MRSA 6	WBF	** + **	** − **	** + **
MRSA 7	WBF	** + **	** − **	** − **
MRSA 8	WBF	** + **	** − **	** + **
MRSA 9	WBF	** + **	** + **	** + **
MRSA 10	WBF	** + **	** − **	** − **
MRSA 11	SBF	** + **	** − **	** − **
MRSA 12	WBF	** + **	** − **	** + **
MRSA 13	WBF	** + **	** + **	** + **
MRSA 14	SBF	** + **	** + **	** + **
MRSA 15	SBF	** + **	** − **	** + **
MRSA 16	SBF	** + **	** + **	** + **
MRSA 17	SBF	** + **	** − **	** − **
MRSA 18	WBF	** + **	** − **	** + **
MRSA 19	WBF	** + **	** − **	** + **
MRSA 20	WBF	** + **	** − **	** − **
MRSA 21	WBF	** + **	** + **	** − **
MRSA 22	SBF	** + **	** + **	** + **
MRSA 23	MBF	** + **	** − **	** − **
MRSA 24	SBF	** + **	** + **	** + **
MRSA 25	SBF	** + **	** + **	** + **
MRSA 26	MBF	** + **	** + **	** + **
MRSA 27	SBF	** + **	** + **	** + **
MRSA 28	WBF	** + **	** − **	** + **
MRSA 29	SBF	** + **	** − **	** − **
MRSA 30	SBF	** + **	** + **	** + **

### Antibacterial activity of PCN and MICs

**Fig. 1:**
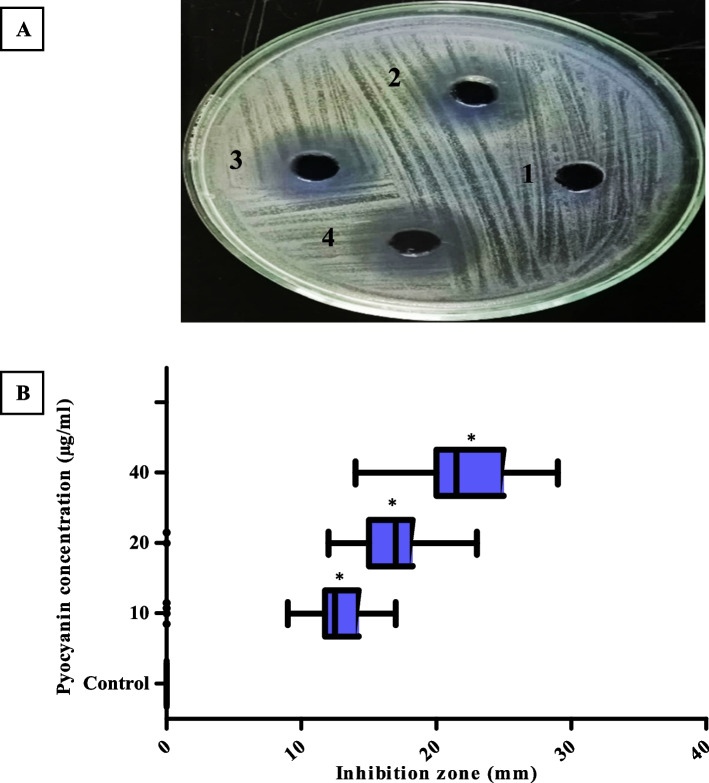
**A** The corresponding zone of inhibition produced by PCN (2, 3, and 4) around wells against MRSA at a concentration of 40 µg/ml, SDW was a negative control (1). **B** The dose-dependent antibacterial activity produced by PCN against MRSA isolates at concentrations of 40, 20, and 10 µg/ml. The error bars indicate standard deviations. The asterisks represent statistical significance (*P* < 0.05)

The antimicrobial activity of PCN was examined using the well-diffusion method against MRSA strains. The results showed that PCN exhibited a significant (*P* < 0.05) dose-dependent antibacterial activity against MRSA with inhibition zones ranging from (15-36 mm), (13-30 mm), and (10-19 mm) at concentrations of 40, 20, and 10 µg/ml, respectively, as shown in Fig. 1. The MICs of PCN against the tested MRSA isolates (*n* = 30) were also determined by the microdilution technique, and all strains had the same MIC value of 8 μg/ml.

### Biofilm eradication activity of PCN

#### Crystal violet assay for determination of biofilm mass

**Fig. 2:**
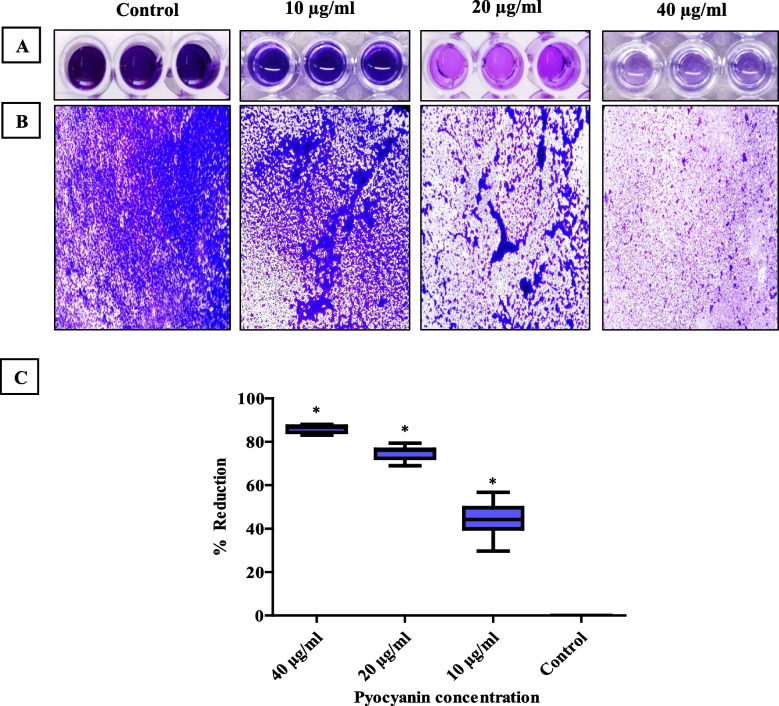
Concentration-dependent eradication of MRSA strains (*n* = 10) pre-established biofilms after PCN treatment using the crystal violet assay. **A** The corresponding crystal violet-stained biofilm of MRSA after PCN treatment at different concentrations in a microtiter plate. **B** Micrographs of the disrupted biofilm of MRSA after PCN treatment at different concentrations on the glass surface by LM. **C** The scatter plot indicates the percentage of reduction in biofilm mass of MRSA strains after PCN treatment at concentrations of 40, 20, and 10 µg/ml. SDW served as a negative control. The error bars indicate standard deviations. The asterisks represent statistical significance (*P* < 0.05)

Pre-established (48 h) MRSA biofilms were treated with PCN at different concentrations (40, 20, and 10 µg/ml) using a microtiter plate assay. PCN treatment led to a significant eradication (*P* < 0.05) of pre-established biofilm by (83–88%), (69–79.4%) and (29.7–56.8%) for 40, 20, and 10 µg/ml of PCN, respectively, as shown in Figs. 2A, C. The reduction in biomass of pre-established biofilm after PCN treatment was confirmed using LM, as shown in Fig. 2B.

#### Counting of CFU and EPS quantification

**Fig. 3:**
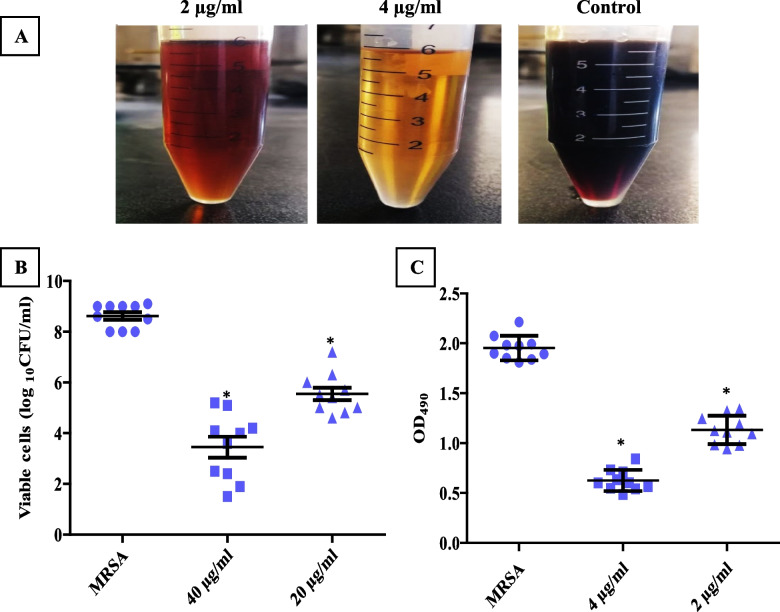
Illustration of the biofilm eradication mechanism of PCN against MRSA isolates (*n* = 10). **A** and** C** The reduction in EPS production after PCN treatment at 1/2 and 1/4 MICs of PCN was determined by the phenol–sulfuric acid method. **B** The effect of PCN on bacterial viability within a pre-established MRSA biofilm shows a reduction in bacterial viability after treatment with 40 and 20 µg/ml of PCN. The error bars indicate standard deviations. The asterisks represent statistical significance (*P* < 0.05)

To understand how PCN could eradicate the pre-established MRSA biofilm, we assessed the effect of PCN treatment on bacterial viability within the biofilm by determining the viable bacterial count as well as the effect of PCN on EPS, the main matrix component, using the phenol–sulfuric acid method. We found that the bacterial viability significantly decreased (*P* < 0.05) after PCN treatment, as shown in Fig. 3B, and the EPS significantly decreased (*P* < 0.05) after PCN treatment, as shown in Figs. 3A, C.

#### Confocal laser scanning microscopy analysis

**Fig. 4:**
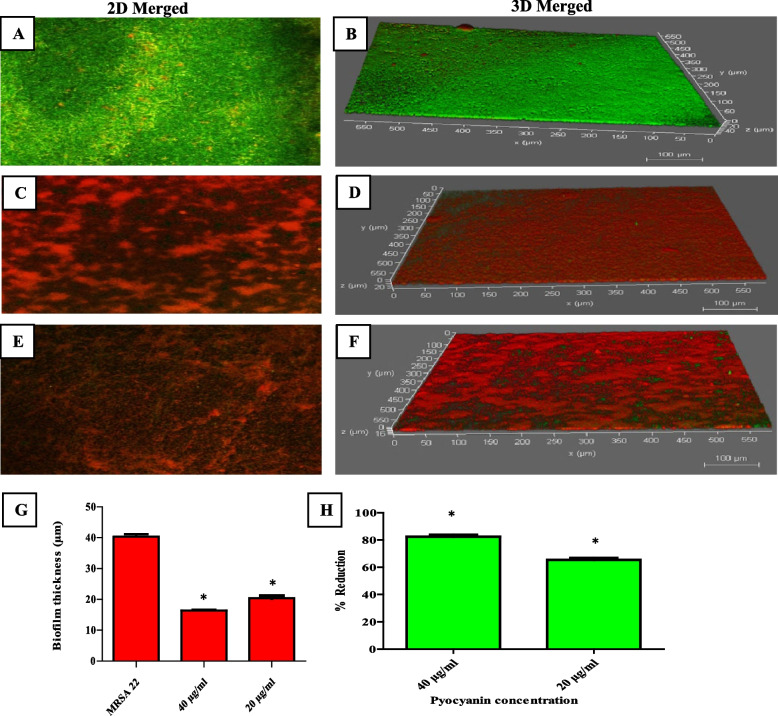
The effect of PCN on bacterial viability (2D merged) and biofilm thickness (3D merged) of a pre-established MRSA biofilm was assessed by CLSM. **A**, **B** Untreated MRSA biofilm. PCN-treated MRSA biofilm at concentrations of 20 (**C**, **D**) and 40 µg/ml (**E**, **F**). **G** Biofilm thickness before and after PCN treatment. **H** The percentage of reduction in bacterial viability after PCN treatment. The error bars indicate standard deviations. The asterisks represent statistical significance (*P* < *0.05*)

The CLSM was used for further investigation of the antibiofilm activity of PCN against MRSA isolates. After PCN treatment of a 48-h pre-established MRSA biofilm, the viability and the biofilm thickness were visualized using acridine orange and propidium iodide for staining dead cells (red) and viable cells (green). PCN penetrated the biofilm matrix and significantly reduced (*P* < 0.05) the viability of bacterial cells by approximately 65 and 82% for 20 and 40 µg/ml of PCN, respectively, as shown in Fig. 4. Moreover, the thickness of the biofilm matrix was significantly reduced (*P* < 0.05) by 50 and 60% for 20 and 40 µg/ml of PCN, respectively, as shown in Fig. 4.

#### Scanning electron microscopy analysis

**Fig. 5:**
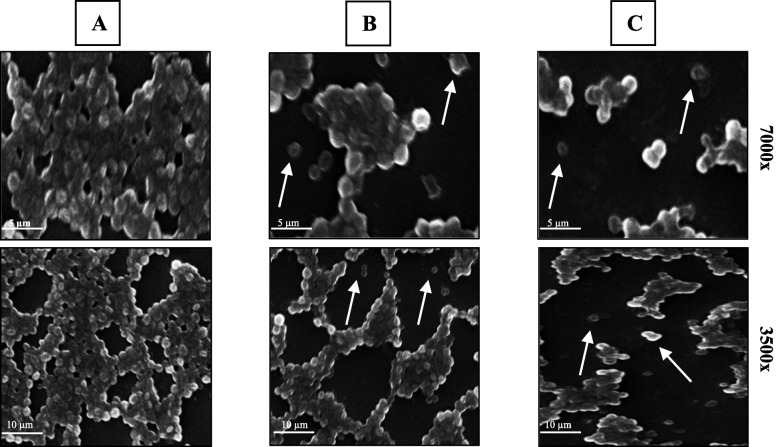
Disruption of a pre-established MRSA biofilm after PCN treatment. **A** The biomass of MRSA biofilm shows a well-established biofilm architecture. Reduction in the biomass of MRSA biofilm and loss of the strong connection between bacterial cells after PCN treatment at 20 µg/ml (**B**) and 40 µg/ml (**C**). The white arrows show the detached bacterial cell after PCN treatment

The ability of PCN to penetrate the biofilm matrix and exert its antibacterial activity on the bacterial cells within the matrix was further confirmed using SEM. Results revealed that the MRSA biofilm was disrupted after PCN treatment at a concentration of 20 and 40 µg/ml. The number of bacterial cells decreased in the treated pre-established MRSA biofilm compared to the untreated pre-established MRSA biofilm. The connection between bacterial cells was disrupted after PCN treatment, and the biofilm architecture was destroyed compared to an untreated, pre-established MRSA biofilm, as shown in Fig. 5.

#### Effect of PCN on growth curve

**Fig. 6:**
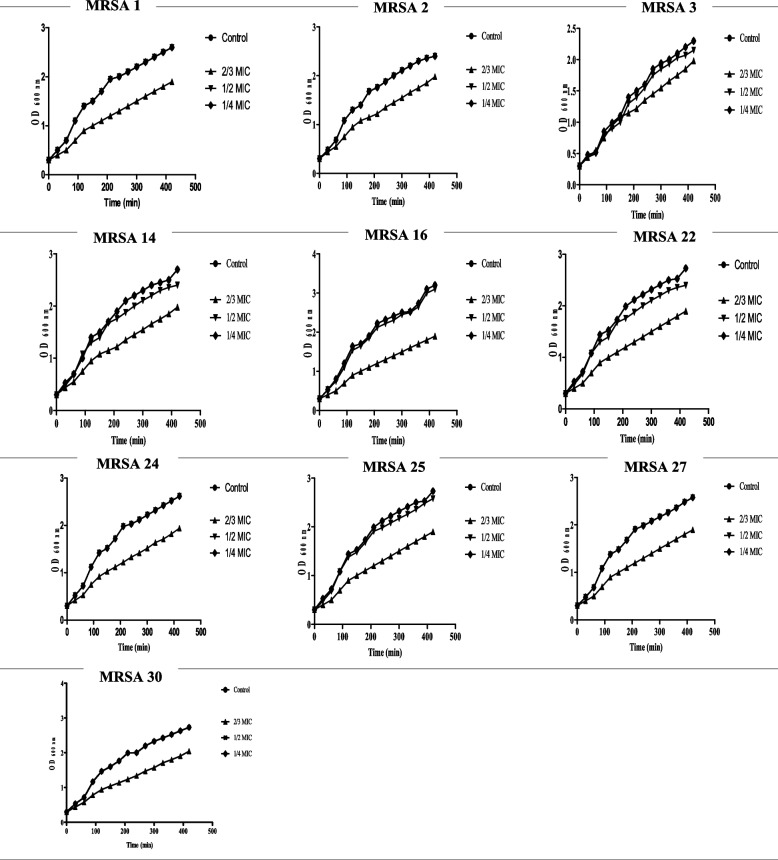
Growth curve of MRSA isolates (*n* = 10) at 30-min time intervals that were grown in the absence and presence of 2/3, 1/2, and 1/4 MICs of PCN. The results were the average of three representative replicates

The growth of MRSA isolates no. 1, 2, 3, 14, 16, 22, 24, 25, 27, and 30 with 2/3, 1/2, and 1/4 MIC of PCN and without PCN was measured at OD 600, as shown in Fig. 6. According to the obtained growth curves, 1/2 and 1/4 MICs of PCN had negligible effects on the growth of MRSA isolates, and so we selected these concentrations for further study. On the other hand, 2/3 of the MIC of PCN affected the growth rate of MRSA isolates.

#### Virulence attenuation after PCN treatment

**Fig. 7:**
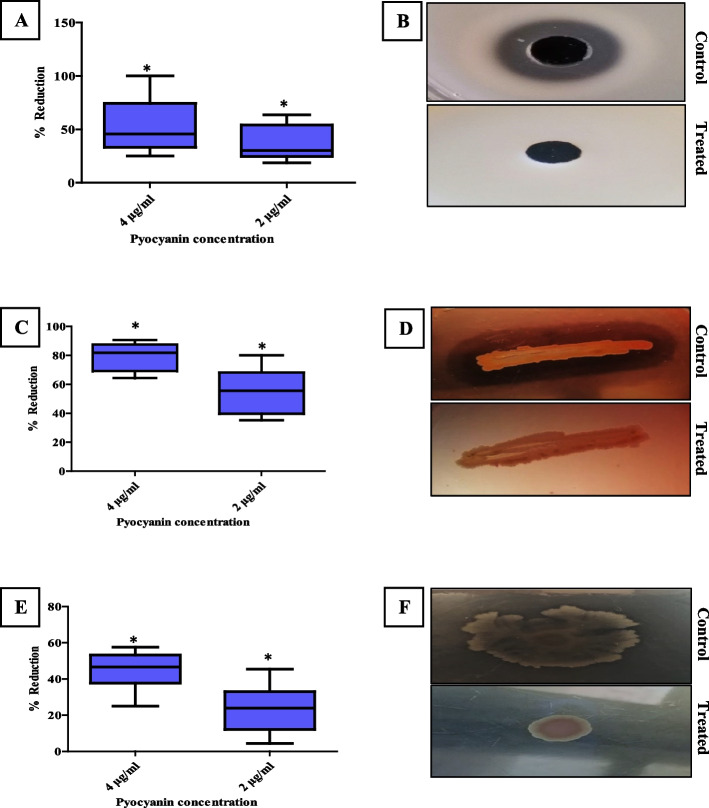
Attenuation of the virulence factors of MRSA strains (*n* = 10) after PCN treatment. The percentage of reduction in the proteolytic activity (**A**), hemolytic activity (**C**), and motility (**E**) of MRSA strains by 4 and 2 µg/ml of PCN. **B** No zone of clearance (no proteolytic activity) was detected after PCN treatment. **D** No visible clear zone was detected around the bacterial growth (no hemolytic activity) after PCN treatment. **F** The treated MRSA strain remained at the inoculation site (no motility). The error bars indicate standard deviations. The asterisks represent statistical significance (*P* < 0.05)

The effect of PCN on virulence factors of MRSA isolates, such as proteolytic activity, hemolytic activity, and motility, was assessed. The proteolytic activity of MRSA isolates was estimated in the absence and presence of PCN using skimmed milk plates. Untreated MRSA showed a zone of clearance, while treated MRSA showed no zone of clearance (no proteolytic activity), as shown in Fig. 7B. Proteolytic activity of MRSA strains was significantly (*P* < *0.05*) inhibited by (25–100%) and (18.8–63.6%) for 4 and 2 µg/ml of PCN, respectively, as shown in Fig. 7A. The hemolytic activity of MRSA isolates was estimated in the absence and presence of PCN using blood agar plates. PCN showed a strong inhibition in the hemolytic activity of MRSA isolates; untreated MRSA showed a visible clear zone detected around the bacterial growth, while PCN inhibits hemolytic activity and no visible clear zone was detected around the bacterial growth, as shown in Fig. 7D. Hemolytic activity was significantly (*P* < *0.05*) inhibited, using a spectrophotometric assay, by (64.3–90.6%) and (35.3–80.1%) for 4 and 2 µg/ml of PCN, respectively, as shown in Fig. 7C. The motility of MRSA strains was estimated in the absence and presence of PCN using a motility plate. Untreated MRSA isolates spread away from the site of inoculation (diffuse zone), while treated MRSA isolates remained at the inoculation site (no motility), as shown in Fig. 7F. The motility was significantly (*P* < *0.05*) inhibited by (25–57.6%) and (4.4–45.5%) for 4 and 2 µg/ml of PCN, respectively, as shown in Fig. 7E.

#### Effect of PCN on accessory gene regulator A (*agrA* Gene)

**Fig. 8:**
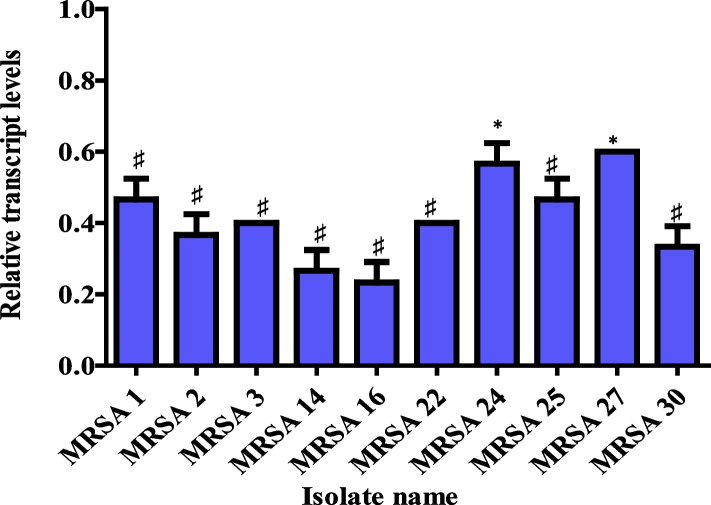
Relative transcript levels in the *agrA* gene after PCN treatment. The error bars indicate standard deviations. ♯ means the change in the *agrA* gene expression is two-fold or more. The asterisks mean the change in *agrA* gene expression is more than one-fold but less than two-fold

The Agr QS is considered a universal regulator of *S. aureus* virulence factors. Therefore, the effect of PCN on the Agr QS of MRSA strains was examined using qRT-PCR by studying the expression levels of the *agrA* gene after PCN treatment (4 µg/ml) in the most virulent MRSA isolates (n = 10). The expression of the *agrA* gene significantly (*P* < *0.05*) decreased after PCN treatment in all tested MRSA strains, as shown in Fig. 8. The percentages of reduction in the *agrA* gene expression levels were 53.3, 63.3, 60, 73.3, 76.67, 60, 43.3, 53.3, 40, and 66.67% for MRSA strains Nos. 1, 2, 3, 14, 16, 22, 24, 25, 27, and 30, respectively.

#### In silico interaction of PCN and AgrA protein

**Fig. 9:**
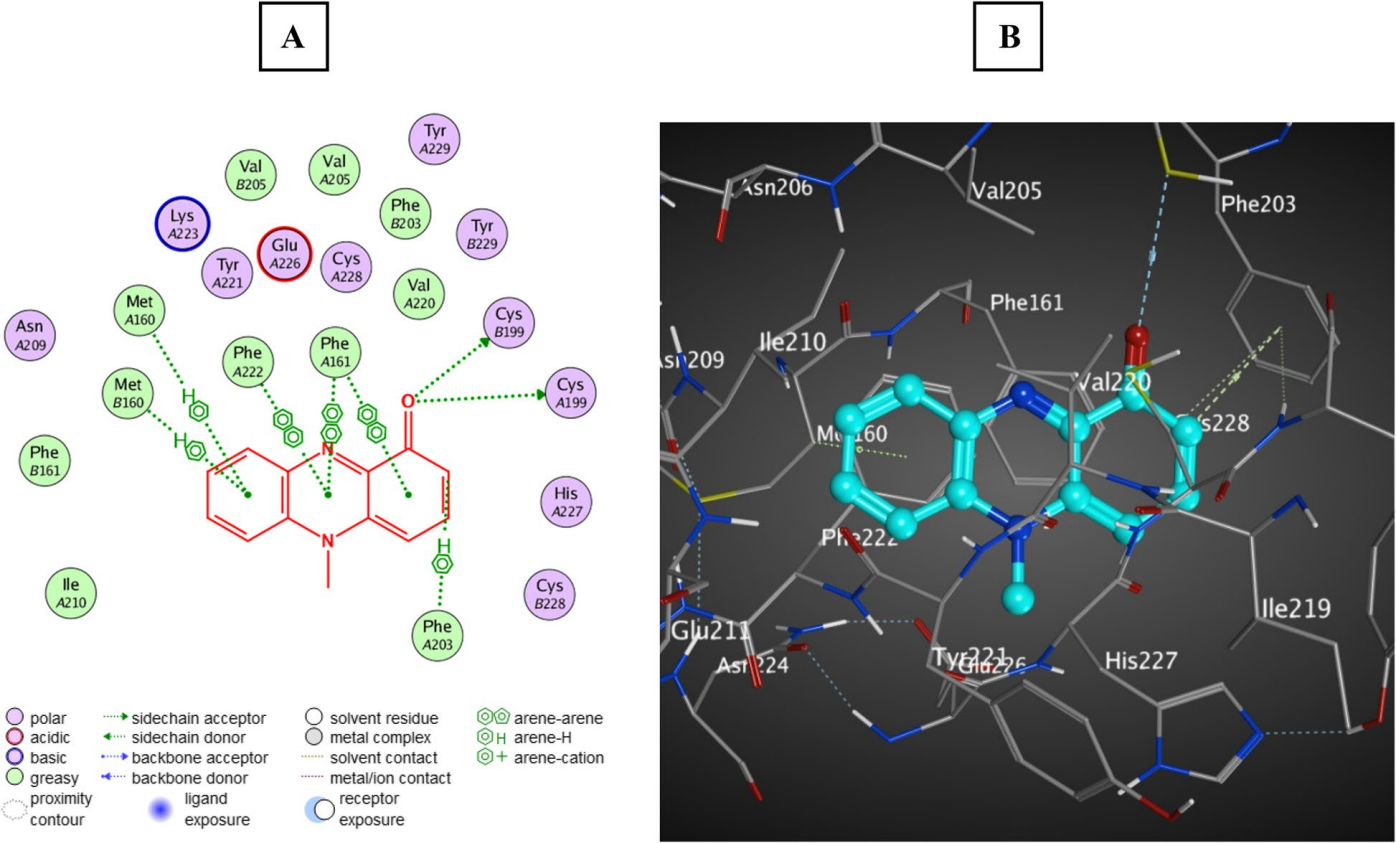
Docking of PCN within the active site of AgrA protein (PDB ID: 3BS1). **A** The 2D binding mode of PCN with AgrA protein. **B** The 3D binding mode of PCN with AgrA protein (the PCN is colored in cyan)

Based on our results, PCN phenotypically decreased Agr QS-dependent virulence factors, such as protease, hemolysin, and motility. Additionally, the expression level of the *agrA* gene decreased at the molecular level. To gain a better understanding of the plausible mechanism by which PCN can induce its anti-QS activity, we proceeded to examine the interaction of PCN with the main regulator protein of Agr QS (AgrA protein) (PBD ID: 3BS1). Molecular docking was accomplished in standard precision mode using the glide ligand-docking module. Results showed that PCN was appropriately oriented in the active site of the AgrA protein with a significant docking score (− 10 kcal Mol − 1); PCN made a strong complex with the AgrA protein through Met 160, Phe 222, Phe 161, Cys 199, and Phe 203 amino acids, as shown in Fig. 9. Therefore, we found that PCN could block Agr QS by interacting with the main regulator protein AgrA, preventing its regulatory action in the production of *S. aureus* virulence factors and genes.

#### Healing of infected skin wound

**Fig. 10:**
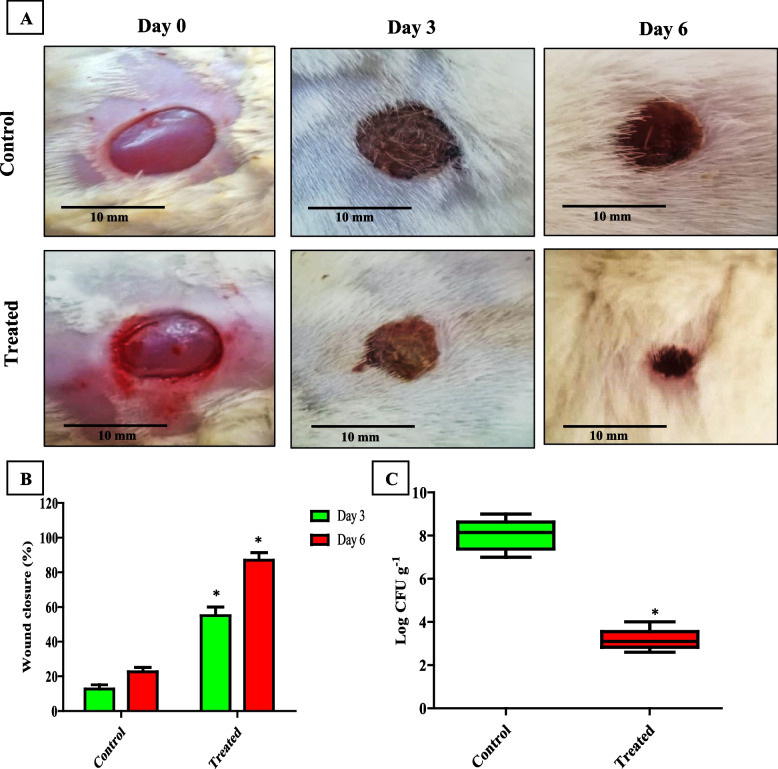
The effect of PCN in controlling MRSA skin wound infection. **A** Photographs of representative rats from each group (treated with vehicle vs. PCN). **B** Percentage of wound closure with and without PCN treatment. **C** Mean log wound bacterial count in PCN-treated and untreated MRSA-infected rats. The error bars indicate standard deviations. The asterisks represent statistical significance (*P* < 0.05)

The effect of PCN on MRSA infection was investigated using the rat wound infection model by measuring the wound closure in MRSA male rats with and without PCN treatment. The percentage of wound closure was significantly (*P* < *0.05*) higher in treated rats compared to untreated rats on different days of the experiment (Table 1, S1). In both the untreated and treated groups, it was observed that the increase in the percentage of wound closure was time-dependent, as shown in Fig. 10A, B. The efficacy of PCN was further investigated by analyzing the bacterial burden in rat-infected skin wounds. The treated wounds showed a significantly (*P* < *0.05*) lower bacterial burden (mean log CFU̸ g approximately equal to 8) than the untreated wounds (mean log CFU̸ g approximately equal to 3) after 6 days of inoculation, as shown in Fig. 10C.

#### Histopathological examination of skin wound

**Fig. 11:**
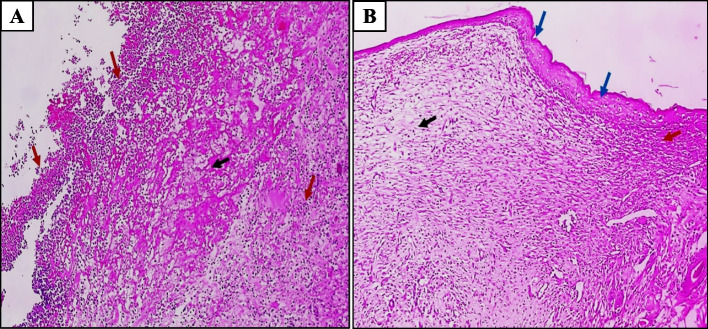
Representative images of H&E histological sections of the skin tissue of male rats sacrificed after 6 days. **A** A section in the skin of MRSA-infected and untreated rats showed skin ulceration covered by heavy inflammation (acute and chronic inflammatory cells) (red arrows) with necrotic debris (black arrow) but no epithelization [H&E × 100]. **B** A section in the skin of MRSA-infected and PCN-treated rats showed complete epithelization (blue arrows) with underlying granulation tissue (black arrow) and few inflammatory cells (red arrow) [H&E × 100]

The wound-healing capacity of PCN was further assessed by H & E. After the excision of wounds and staining, the stained sections were utilized to evaluate the effect of PCN on the healing of tissue. In the MRSA-infected and untreated group (control group), the histopathological examination of tissues showed wide-ranging destruction, skin ulceration that was covered by heavy inflammation (acute and chronic inflammatory cells), and necrotic debris with no epithelization, as shown in Fig. 11A. In the MRSA-infected and PCN-treated group (the treated group), the histopathology examination showed complete epithelization with underlying granulation tissue and few inflammatory cells, as shown in Fig. 11B.

## Discussion

The WHO has urged the scientific community to search for new approaches to combat antibiotic resistance, a dangerous public health problem as more and more bacterial pathogens have developed resistance to antibiotics and the present treatment options have become few and ineffective [43–45]. Numerous issues lead to the occurrence of bacterial resistance, such as biofilm formation [46, 47]. Biofilm acts as a physical barrier, preventing the penetration of antibiotics and their interaction with bacterial cells, and protecting bacteria from innumerable environmental pressures [9]. Moreover, bacterial cells in their biofilm mode established an up to 1000-fold lower susceptibility to most antibiotics compared to their planktonic mode [48, 49]. Hence, biofilm-associated infections are exceptionally complicated to treat with common antibiotics, leading to chronic infections and non-healing wounds [50]. Therefore, it has been necessary to find new antibacterial agents that are effective against biofilm.

The great advantages of natural pigments, such as biodegradability and safety, compared to synthetic pigments, have captured the attention of the industry in human applications [50]. Despite the availability of many natural pigments, the most preferred natural pigments are microbial pigments because of their simple and rapid pigment extraction and scaling up. The safety of bacterial pigments, such as carotenoids, prodigiosin, rhodopsins, pyoverdine, and PCN, activates their use in the pharmaceutical, food, cosmetic, and textile industries [51–53]. The therapeutic behavior of bacterial pigments is linked to their antioxidant, anticancer, cytotoxic, and antimicrobial properties [52]. Consequently, the potential antibacterial, antibiofilm, and anti-QS activities of *P. aeruginosa*-derived PCN against MRSA were assessed in the present study.

According to our study, PCN showed a high killing power against MRSA isolates with a MIC value equal to 8 µg/ml. In agreement with our study, El-Fouly et al. [54] observed the growth inhibition activity of PCN against *S. aureus*, *Escherichia coli*, *Klebsiella* species, *Salmonella typhi*, and *Shigella* species. Aziz et al. found that PCN inhibited the growth of *S. aureus*, *K. pneumoniae*, *Enterococcus faecalis*, *Burkholderia cepacia*, and *E. coli* [55]. Additionally, according to Hamad et al. [56], PCN exerted antibacterial activity against *Bacillus* cereus, *S. aureus*, *Staphylococcus sciuri*, *E. coli*, *S. typhi*, *Salmonella enterica*, *K. pneumoniae*, and *Lactococcus lactis*. Researchers consider that the key mechanism by which PCN exerts its antibacterial activity is that PCN simply permeates cell membranes and accepts electrons from NADH or NADPH. Under aerobic conditions, PCN passes those electrons to O_2_, and reactive oxygen species (ROS) are created, which leads to oxidative stress and the inhibition of the ion's interaction with the membrane, respiration, and the active transport of solutes; consequently, the bacterial growth is inhibited [17–19].

Besides affecting the MRSA isolates in their planktonic mode, we found that PCN inhibited the growth of MRSA isolates in their biofilm mode. The crystal violet assay showed that PCN significantly eradicated biofilms of tested MRSA isolates, and the mechanism by which PCN eradicated pre-established MRSA biofilms was associated with decreasing bacterial viability and reduction of the EPS matrix. The reduction of the viability of bacterial cells and biofilm thickness by PCN treatment was further confirmed using CLSM. Moreover, SEM images proved that PCN disrupted MRSA biofilms, and the biomass of the MRSA biofilm was decreased after PCN treatment. In accordance with our study, PCN extracted from *P. aeruginosa* BTRY1 exhibited a remarkable reduction in foodborne pathogens’ biofilm formation, belonging to the genera *Bacillus*, *Staphylococcus*, *Brevibacterium*, and *Micrococcus* [57]. Additionally, Saleem et al. reported that PCN inhibited biofilm formation and disrupted pre-formed biofilms by *B. cereus*, *S. aureus*, and *K. pneumoniae* [58].

The pathogenicity of *S. aureus* largely depends on the production of plentiful extracellular virulence factors (e.g., hemolysin and enterotoxins). Subsequently, an alternate strategy in the treatment of *S. aureus* is targeting bacterial virulence factors [35]. In *S. aureus*, the majority of virulence factors are regulated by QS, which is under the control of the agr (accessory gene regulatory) operon [59]. The expression of the agr operon is subject to transcriptional regulation by the AgrA response regulator [59]. Upon AgrA phosphorylation, it activates the RNAIII and RNAII promoters, in addition to several other transcriptional targets [60]. The RNAII segment of agr is an operon of four genes, *agrBDCA*, which encode all the main components of QS [61]. Blocking the Agr QS system leads to the downregulation of many virulence genes and the reduction of Agr QS-dependent phenotypes [3, 11, 62].

In our study, PCN inhibited the *agrA* gene expression by 53.3, 63.3, 60, 73.3, 76.67, 60, 43.3, 53.3, 40, and 66.67% for MRSA strains Nos. 1, 2, 3, 14, 16, 22, 24, 25, 27, and 30, respectively. Additionally, the anti-QS activity of PCN was proven through diminished hemolytic activity, proteolytic activity, and motility of MRSA isolates after PCN treatment with 1/2 and 1/4 MICs of PCN without affecting bacterial viability. In addition, the in silico analysis revealed that PCN binds to the AgrA protein at a critical location that is required for proper folding, DNA binding, and exerting its QS regulation activity [63]. In agreement with our study, the interaction of salicylic acid with the AgrA protein led to the downregulation of the *agrA* gene and the reduction of the hemolytic and proteolytic activities of *S. aureus* [62]. Blocking the activity of AgrA protein by Azan-7 resulted in a significant reduction in the expression of *hla*, *psm*α, *hysA*, *agrA*, *cap1A*, and *cap1C* genes and Agr QS-dependent virulence such as hemolysin [3]. Additionally, staquorsin showed its potent inhibition of the Agr QS system by blocking AgrA protein activity via inhibition of hemolysin and lipase production as well as a reduction in the effector transcript RNA III [11]. Furthermore, the number of amino acid residues involved in PCN binding with AgrA protein is greater than those involved in previously published AgrA-dependent QS inhibitors such as salicylic acid, Azan-7, and staquorsin, confirming its strong activity [3, 11, 62].

For further confirmation of the ability of PCN to compete with MRSA infection, we assessed the effect of PCN on the wound infection caused by virulent MRSA isolates, which is one of the most common bacteria isolated from chronic wounds [64, 65]. We found that PCN was able to accelerate the wound-healing process and reduce the level of inflammation in the tissues. Our study is in agreement with the studies that discussed the anti-inflammatory action of PCN; Allen et al. reported that the production of IL-6, IL-1b, keratinocyte-derived chemokine, and macrophage inflammatory protein (MIP)-2 decreased in the lungs of C57BL/6 mice infected by *P. aeruginosa* by PCN [66]. Furthermore, Fujihara et al. found that PCN reduced the levels of TNF-a and IL-1b [67]. Marreiro de Sales-Neto et al. discussed that PCN exerted its anti-inflammatory activity in LPS-activated macrophages by reducing the generation of nitric oxide, IL-1b, and TNF-a without causing macrophage mortality [68].

In conclusion, *P. aeruginosa*-derived PCN showed remarkable antibacterial and antibiofilm activities against MRSA as well as an anti-QS effect through binding to the AgrA protein. Hence, PCN is considered a promising antibacterial candidate for the treatment of biofilm-associated MRSA infections as well as a QS attenuation agent through blocking AgrA protein activity. Further studies are required to assess the effect of PCN in combination with antibiotics in the treatment of bacterial infections.

## Supplementary Information

**Additional file 1:** **Supplementary data Table 1.** The percentage of wound closure with and without pyocyanin treatment after 3 and 6 days of infection. The green column represents the size of the first wound, and the red column represents the size of the second wound in the same rat (each rat had two excisional wounds).

## Data Availability

All data generated or analyzed during this study are included in this published article.
