# Supplementary material for: Antibacterial, antibiofilm, and anti-quorum sensing activities of pyocyanin against methicillin-resistant Staphylococcus aureus: in vitro and in vivo study
Source: BMC Microbiol. 2023 Apr 24;23:116. doi: 10.1186/s12866-023-02861-6 (PMC10124065; doi:10.1186/s12866-023-02861-6)
Supplement: Supplementary file 1 — Additional file 1: Supplementary data Table 1. The percentage of wound closure with and without pyocyanin treatment after 3 and 6 days of infection. The green column represents the size of the first wound, and the red column represents the size of the second wound in the same rat (each rat had two excisional wounds). [file 12866_2023_2861_MOESM1_ESM.docx]

**Supplementary data (Table. 1)**

The percentage of wound closure with and without pyocyanin treatment after 3 and 6 days of infection. The green column represents the size of the first wound, and the red column represents the size of the second wound in the same rat (each rat had two excisional wounds).

| **Treated** | | | | | | **Control** | | | | | | **Rats** |
| --- | --- | --- | --- | --- | --- | --- | --- | --- | --- | --- | --- | --- |
| **Day 6** | | **Day 3** | | **Day 0** | | **Day 6** | | **Day 3** | | **Day 0** | |  |
| 1mm | 1mm | 4mm | 4mm | 10 mm | 10 mm | 8mm | 8mm | 9mm | 9 mm | 10 mm | 10 mm | **1** |
| 1.1mm | 1.1mm | 4.5mm | 4.5mm | 10 mm | 10 mm | 7.5mm | 7.5mm | 8.5mm | 8.5mm | 10 mm | 10 mm | **2** |
| 1.8mm | 1.8mm | 5mm | 5mm | 10 mm | 10 mm | 7.7mm | 7.7mm | 8.7mm | 8.7mm | 10 mm | 10 mm | **3** |
| 1mm | 1mm | 4mm | 4mm | 10 mm | 10 mm | 8mm | 8mm | 9mm | 9mm | 10 mm | 10 mm | **4** |
| 1.1mm | 1.1mm | 4.5mm | 4.5mm | 10 mm | 10 mm | 7.7mm | 7.7mm | 8.7mm | 8.7mm | 10 mm | 10 mm | **5** |
| 1.8mm | 1.8mm | 5mm | 5mm | 10 mm | 10 mm | 7.5mm | 7.5mm | 8.5mm | 8.5mm | 10 mm | 10 mm | **6** |
| **87%** | | **55%** | | **0 %** | | **22.67%** | | **12.67%** | | **0 %** | | **Average % reduction in wound size** |
